# Cisplatin in 5% Ethanol Eradicates Cisplatin-Resistant Lung Tumor by Killing Lung Cancer Side Population (SP) Cells and Non-SP Cells

**DOI:** 10.3389/fgene.2013.00163

**Published:** 2013-08-29

**Authors:** Qi Niu, Wei Wang, Yong Li, Douglas M. Ruden, Qian Li, Fenghua Wang

**Affiliations:** ^1^Department of Medical Oncology, No. 309 People’s Liberation Army HospitalBeijing, People’s Republic of China; ^2^Department of Obstetrics and Gynecology, Institute of Environmental Health Sciences, C.S. Mott Center for Human Health and DevelopmentDetroit, MI, USA; ^3^Department of Internal Medicine, Beijing Language and Culture University HospitalBeijing, People’s Republic of China; ^4^Department of Pathology, No. 309 People’s Liberation Army HospitalBeijing, People’s Republic of China

**Keywords:** ethanol, lung cancer, chemoresistance, side population cells, ABCG2

## Abstract

Cancer side population (SP) cells with cancer stem cell-like properties are thought to be responsible for lung cancer chemotherapy resistance and currently no drug can efficiently target them. Breast cancer resistance protein (BRCP/ABCG2) is a major drug transporter in protecting lung cancer SP cells from cytotoxic agents. We showed that a low concentration of ethanol, which inhibits many membrane proteins, inhibits ABCG2 in lung cancer SP cells. Furthermore, cytotoxic cisplatin (DDP) in 5% (vol/vol) ethanol kills SP plus non-SP cancer cells better than either treatment alone in eradicating chemoresistant lung tumors. We found that 5% ethanol did not reduce ABCG2 protein levels, but significantly reduced ABCG2 protein function by a Hoechst 33342 extrusion assay, an ATPase activity assay, and transmission electron microscopy. Further, DDP in 5% ethanol (5% ethanol–DDP) induced apoptosis of the SP plus non-SP cancer cells both *in vitro* and *in vivo*. In DDP-resistant A549/DDP lung tumor-bearing Balb/C nude mice, intratumoral injection of 5% ethanol–DDP regressed tumors and significantly improved survivals compared with 5% ethanol, DDP alone, or control. Intratumoral injection of 5% ethanol–DDP helped eradicate tumors in 30% (3/10) of the mice after 4 weeks treatment. By killing SP and non-SP cancer cells, 5% ethanol–DDP could eradicate DDP-resistant lung tumor and extend survival, providing a novel way to improve chemoresistant lung cancer survival for clinic.

## INTRODUCTION

Chemotherapy resistance, believed by many to be mediated by cancer stem-like cells, accounts for a majority of lung cancer deaths (Dean et al., 2005; Eramo et al., 2010). The cancer stem cell (CSC) theory states that tumors are organized in a hierarchical manner similar to normal tissues, with a sub-population of tumorigenic stem-like cells that are chemoresistant and generate relapse (Dean et al., 2005). Recent observations found that CSC populations are flexible and in dynamic states as cancer cells can also dedifferentiate into CSCs (Gerlinger et al., 2012; Visvader and Lindeman, 2012).

Side population (SP) cells with CSC-like properties were first identified in leukemia and subsequently isolated from solid tumors, including breast, brain, lung, liver, prostate, colon, pancreatic, and head and neck cancers (Bonnet and Dick, 1997; Eramo et al., 2008; Charafe-Jauffret et al., 2009; Singh et al., 2010).

Side population cells express high levels of breast cancer resistance protein (BRCP/ABCG2) which functions as an ATP-dependent membrane transporter. ABCG2 efficiently effluxes a variety of chemotherapy drugs or drug conjugates in SP cells and is believed to be the main basis of chemotherapy resistance (Singh et al., 2010; Niu et al., 2012; Zhang et al., 2012).

We previously isolated SP cells from cisplatin (DDP)-resistant human lung adenocarcinoma A549/DDP cells by fluorescence activated cell sorting (FACS) and demonstrated that they could be used to screen ways to kill SP cells with CSC-like properties (Niu et al., 2012).

Current investigated CSC-targeting agents including low molecular weight heparin, metformin, dopamine receptor antagonist, mithramycin, salinomycin, sulforaphane, miR-34a and other CSC-specific signaling pathway inhibitors such as molecules affecting Wnt, EpCAM, Hedgehog, and Delta/Notch signaling have been shown effects (Alison et al., 2011; Liu et al., 2011; Li et al., 2012; Niu et al., 2012; Sachlos et al., 2012; Vermeulen et al., 2012; Zhang et al., 2012). However, most of them have only attenuated rather than eradicated solid tumors and could not extend survival *in vivo*. The reason could be the CSCs population is fluid and current CSCs-targeting agents have been designed only to target fixed population (Vermeulen et al., 2012; Visvader and Lindeman, 2012).

Recently, liposomal cisplatin has been formulated and tested thoroughly in preclinical and clinical trials. Experiments in animals showed that liposomal formulation is less toxic than cisplatin. However it did not enhance clinical efficacy (Stathopoulos, 2010; Zisman et al., 2011).

We hypothesize a low concentration of ethanol, which inhibits many membrane proteins, might be able to inhibit ABCG2 in lung SP cells and make them less efficient at pumping out DDP (Lucero et al., 1997; He et al., 2006). Thus, cytotoxic DDP in the low concentration of ethanol may kill lung SP plus non-SP cancer cells and eradicate chemoresistant lung tumor. If so, image-guided intratumoral injection of it might provide a novel way to improve chemoresistant lung cancer survival. Therefore, this study investigates whether 5% ethanol could inhibit ABCG2 and DDP in 5% ethanol could kill lung SP plus non-SP cancer cells *in vitro* as well as its effects on DDP-resistant tumors *in vivo*.

## RESULTS

### SIDE POPULATION SORTING

A SP of 10–18% cells existed in A549/DDP cells. The purity of SP cells was >95% (average 97%) and sorted SP cells were used for further experiments.

### 5% ETHANOL DID NOT AFFECT ABCG2 mRNA EXPRESSION IN SP CELLS AND TUMOR XENOGRAFTS CELLS WITH RT-PCR

**FIGURE 1:**
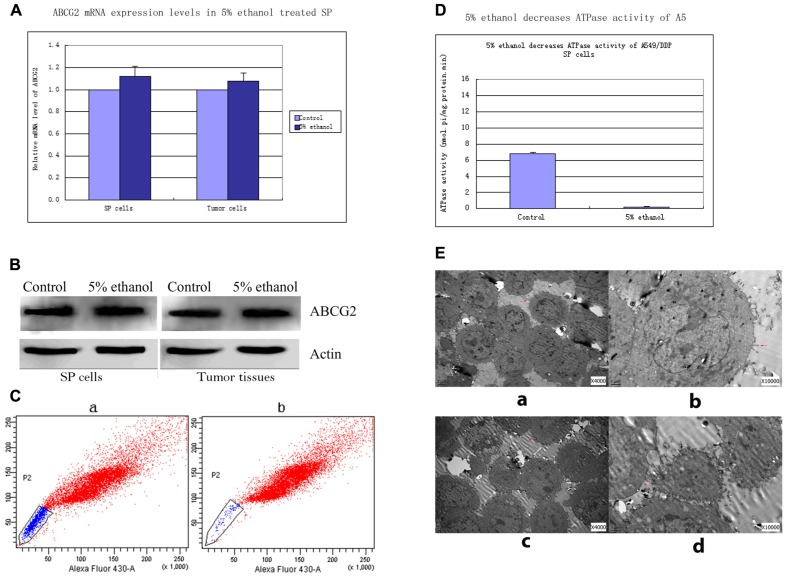
**(A)** ABCG2 mRNA levels in 5% ethanol-treated lung SP cells and tumor cells relevant to controls. Quantitative real time RT-PCR showed that the expressions of ABCG2 mRNA were not significantly changed in SP cells and tumor cells by 5% ethanol treatment compared with its expressions in controls. **(B)** Western blots analysis of ABCG2 expressions in SP cells and tumor tissues treated with 5% ethanol and control. ABCG2 protein expressions in 5% ethanol-treated SP cells and tumor tissues had no significant changes compared with ABCG2 expression in each corresponding control. **(C)** Alteration of ABCG2 protein activity by 5% ethanol. Compared with the side population of 17.45% ± 2.6% in control cells, 5% ethanol significantly decreased the side population by 95.2% (0.83% ± 0.2%). **(D)** 5% ethanol reduced ATPase activity in lung SP cells by ATPase activity assays. Compared with the value of ATPase activity in control SP cells, a significant decrease by 96.6% was observed in the ATPase activity of 5% ethanol-treated SP cells. **(E)** Surface morphology of SP cells treated with 5% ethanol and control by TEM. **(a)** TEM image of control SP cells (×4000). **(b)** TEM image of control SP cells (×10000). **(c)** TEM image of 5% ethanol-treated SP cells (×4000). Notable shortened microvilli and diminution in their number were showed. The shortened microvilli are indicated by arrows. **(d)** TEM image of 5% ethanol-treated SP cells (×10000).

Relative mRNA level of SP cells treated with 5% ethanol to control was 1.12 ± 0.09. Similarly, relative mRNA level of 5% ethanol-treated tumor xenografts cells to control was 1.08 ± 0.07. No significant change was found in ABCG2 mRNA level after 5% ethanol treatment both in SP cells and in xenografts tumor cells (Figure 1A).

### 5% ETHANOL DID NOT CHANGE ABCG2 PROTEIN EXPRESSIONS IN LUNG SP CELLS AND TUMOR TISSUES BY WESTERN BLOTS

ABCG2 protein expressions in 5% ethanol-treated SP cells and tumor tissues had no significant changes compared with ABCG2 expressions in each corresponding control. The values of ABCG2 band density to β-actin band density in control SP cells and 5% ethanol-treated SP cells were 1.06 ± 0.21 vs. 1.10 ± 0.19, *p* > 0.05. The values of ABCG2 band density to β-actin band density in control tumor tissues and 5% ethanol-treated tumor tissues were 1.09 ± 0.16 vs. 1.12 ± 0.20, *p* > 0.05. (Figure 1B).

### ALTERATION OF ABCG2 PUMP ACTIVITY BY 5% ETHANOL

Compared with the sorted SP of 17.45% ± 2.6% by ABCG2 pump in control cells, 5% ethanol significantly decreased the sorted SP by 95.2% (0.83% ± 0.2%; Figure 1C).

### ATPase ACTIVITY ASSAYS

Compared with ATPase activity in control SP cells (6.842 ± 0.462 nmol Pi/mg protein/min), 5% ethanol significantly decreased ATPase activity of SP cells to 0.231 ± 0.091 nmol pi/mg protein.min, by 96.6% (Figure 1D).

### TRANSMISSION ELECTRON MICROSCOPY STUDIES

Three significant changes in SP cells after 5% ethanol treatment were observed with transmission electron microscope (Figure 1E). Firstly, the microvilli of SP cells treated by 5% ethanol were shortened and greatly reduced in number, which reduced the cell membrane surface, thus reducing the number of functional ABCG2 pump in reduced cell membrane surface. Secondly, the mitochondrial, which generates most of ATP as a membrane-enclosed organelle, became swelling and its cristae disappeared. This could make the ABCG2 pump, which is an ATPase, partially inactivated or even totally. Thirdly, the rough endoplasmic reticulum, which is involved in the synthesis of proteins, became swelling and ribosomes fell off from its surface. These could cause disfunction in synthesis of proteins including ABCG2.

### 5% ETHANOL–DDP INDUCED SP CELLS AND NON-SP CELLS APOPTOSIS BY FACS

**FIGURE 2:**
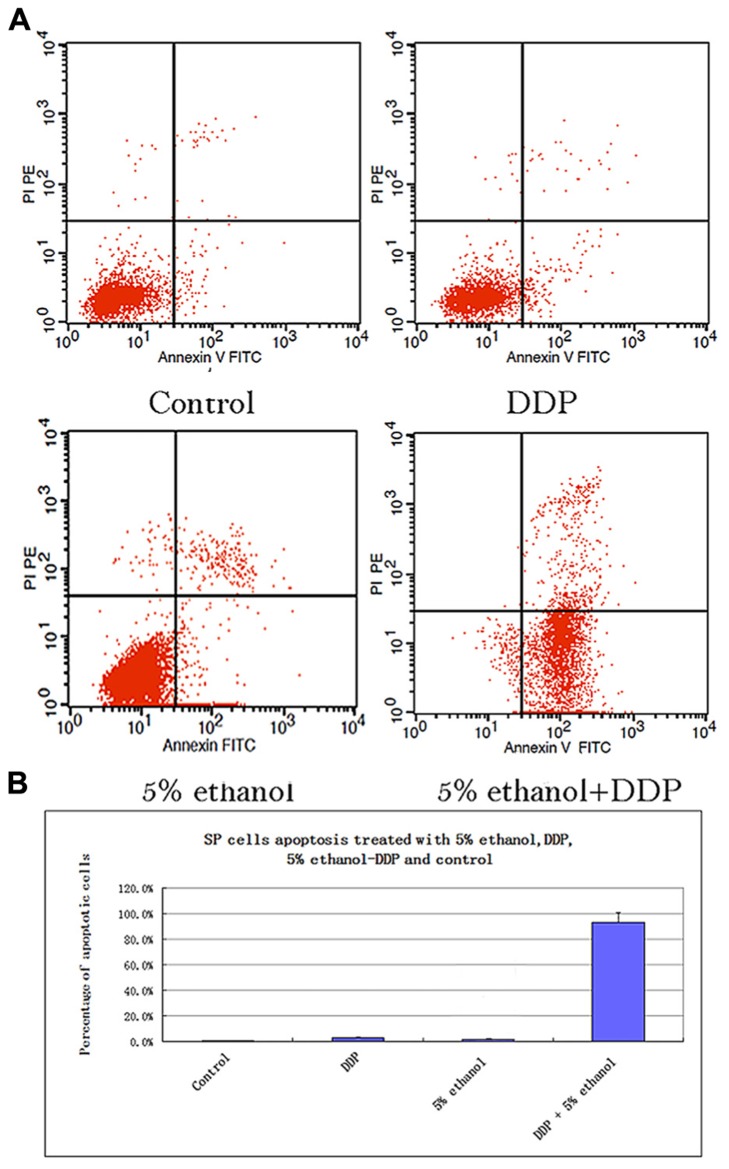
**(A)** Apoptosis analysis of SP cells treated with 5% ethanol, DDP, 5% ethanol–DDP, and control by FACS. **(a)** Apoptosis of control SP cells. **(b)** Apoptosis of 5% ethanol-treated SP cells. **(c)** Apoptosis of DDP-treated SP cells. **(d)** Apoptosis of 5% ethanol–DDP-treated SP cells. **(B)** Apoptosis rate of treated SP cells. The most significant apoptosis rate was found in 5% ethanol–DDP-treated SP cells.

The SP cells apoptosis rates in different treatment groups were: 0.76% ± 0.1% for control, 3.03% ± 0.5% for DDP, 1.86% ± 0.2% for 5% ethanol, and 93.32% ± 7.63% for 5% ethanol–DDP (Figure 2A). Compared with control, 5% ethanol–DDP induced SP cells apoptosis significantly (93.32% ± 7.63% vs. 0.76% ± 0.1%, *p* < 0.05; Figure 2B). In non-SP cells, both 5% ethanol–DDP and DDP induced apoptosis significantly (98.32% ± 0.8% for 5% ethanol–DDP vs. 4.5% ± 0.5% for control, *p* < 0.05; 60.16% ± 6.8% for DDP vs. 4.5% ± 0.5% for control, *p* < 0.05), but 5% ethanol did not (6.4% ± 0.5% for 5% ethanol vs. 4.5% ± 0.5% for control, *p* > 0.05) compared with control.

### 5% ETHANOL–DDP INDUCED TUMOR CELLS APOPTOSIS BY TUNEL

**FIGURE 3:**
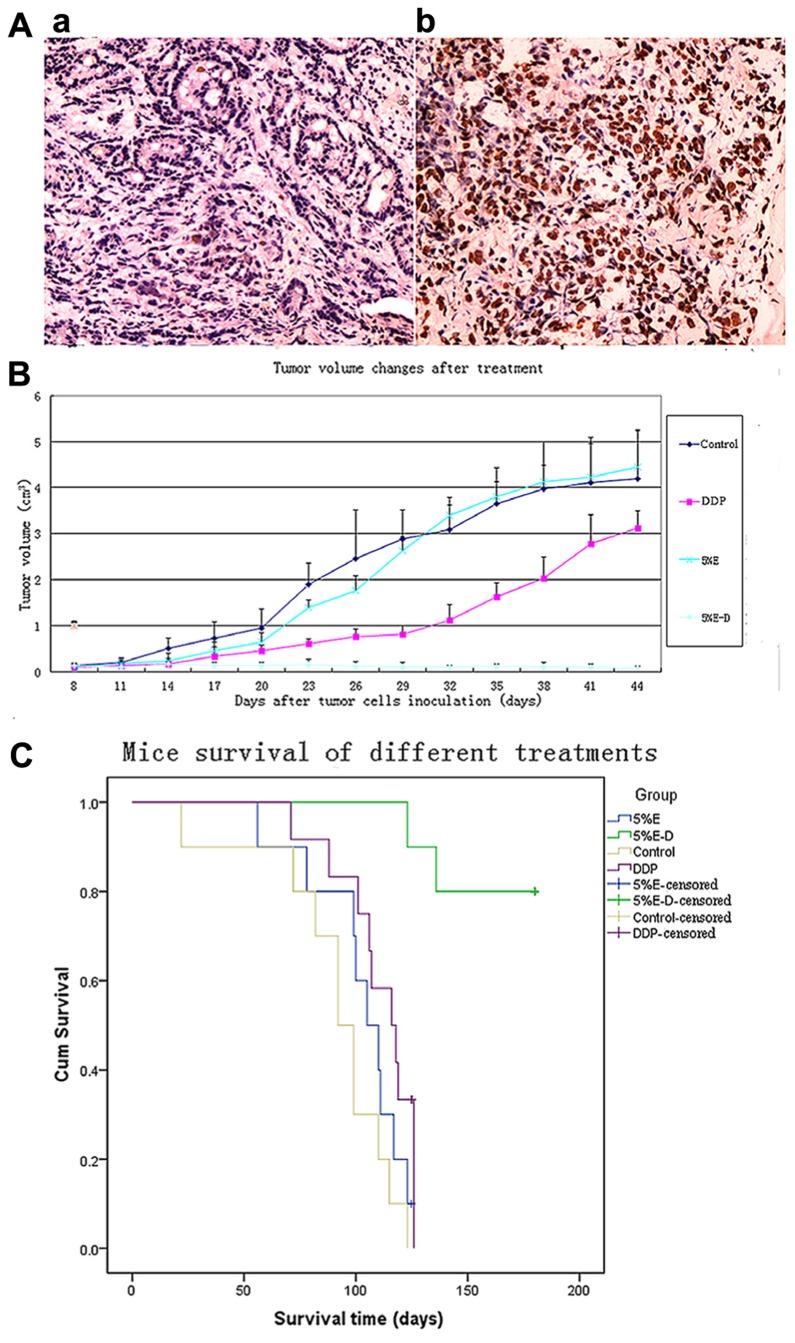
**(A)** Apoptosis analysis of 5% ethanol–DDP-treated tumor tissues with TUNEL staining. **(a)** Control tumor tissue. **(b)** 5% ethanol–DDP treated tumor tissue. Compared with control, 5% ethanol–DDP significantlyIn artworks of Figure 3 has no part whereas the parts (A) and (B) explained in the caption. Kindly advise. increased tumor cell apoptosis rate (60.11% ± 7.52% vs. 5.32% ± 1.76%,* p *< 0.05). **(B)** DDP-resistant tumor size changes by various treatments.5% ethanol–DDP significantly inhibited tumor growth compared with control or DDP alone or 5% ethanol after 4 weeks’ treatment. E–D stands for ethanol–cisplatin. **(C)** Survival of A549/DDP tumor-bearing mice in different groups. 5% ethanol–DDP treatment significantly improved estimated mean survival time compared with control or DDP alone or 5% ethanol. E–D stands for ethanol–cisplatin.

Compared with control, 5% ethanol–DDP increased apoptotic cells as shown in Figure 3A. The rates of total apoptotic cells were 60.11% ± 7.52% in the 5% ethanol–DDP-treated tumor tissues and 5.32% ± 1.76% in the control tumor tissues. 5% ethanol–DDP caused an 11.3-fold increase of apoptosis (Figure 3A).

### DDP-RESISTANT TUMOR XENOGRAFTS IN NUDE MICE WERE COMPLETELY DESTROYED BY 5% ETHANOL–DDP TREATMENT

5% Ethanol–DDP could significantly inhibit tumor growth compared with tumor size of 3.68 ± 0.48 cm^3^ for control after 4 weeks’ treatment (0.11 ± 0.06 cm^3^ for 5% ethanol–DDP,* p *< 0.05). Intratumoral injection of 5% ethanol–DDP to A549/DDP tumor-bearing mice resulted in complete tumor regression in 3 of 10 mice (30%) and tumor growth arrest in the rest seven mice (70%) after 4 weeks’ treatment. Average tumor volume in 5% ethanol–DDP mice was significantly reduced compared with DDP alone (0.11 ± 0.06 cm^3^ for 5% ethanol–DDP vs. 1.63 ± 0.31 cm^3 ^for DDP, *p *< 0.05) or 5% ethanol alone (0.11 ± 0.06 cm^3^ for 5% ethanol–DDP vs. 3.81 ± 0.63 cm^3 ^for 5% ethanol, *p *< 0.05) or control 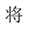 mice (0.11 ± 0.06 cm^3^ for 5% ethanol–DDP vs. 3.68 ± 0.48 cm^3^ for control, *p *< 0.05) after 4 weeks’ treatment. Although 5% ethanol-treated mice have a trend toward increasing tumor size after 3 weeks’ treatment, no significantly difference was found between 5% ethanol-treated mice and control mice after 4 weeks’ treatment (3.81 ± 0.63 cm^3^ for 5% ethanol vs. 3.68 ± 0.48 cm^3^ for controls, *p > *0.05; Figure 3B).

### SURVIVALS OF TUMOR-BEARING NUDE MICE IN DIFFERENT GROUPS

5% Ethanol–DDP treatment could significantly extend survival and it produced the longest survival among all investigated groups of mice. After 180 days’ observance, 80% (8 of 10 mice) of 5% ethanol–DDP-treated mice were still alive with 2 deaths for non-tumor-related reasons, while all 10 control mice died before Day 123. The estimated mean survival time of 5% ethanol–DDP group mice (169.9 ± 6.5 days) was significantly longer compared with estimated mean survival time of DDP or control or 5% ethanol alone groups (110.8 ± 5.1 days for DDP mice, *p *< 0.05; 90.6 ± 9.0 days for control mice, *p *< 0.05; 84.9 ± 4.1 days for 5% ethanol mice, *p *< 0.05). 5% ethanol alone treatment did not significantly change estimated mean survival time compared with control (84.9 ± 4.1 days for 5% ethanol mice, *p *= 0.104; Figure 3C).

## DISCUSSION

In this study, we found that 5% ethanol could significantly inhibit ABCG2 protein function without affecting its mRNA and protein expression in the lung SP cells. 5% ethanol significantly decreased ATPase activity of the lung SP cells by 96.6% with ATPase activity assay and the ABCG2 pump activity by 95.2% with Hoechst 33342 extrusion studies, showing a significant reduction of ABCG2 pump activity. The changes in microvilli, mitochondrial, and rough endoplasmic reticulum of the lung SP cell ultrastructure further confirm ABCG2 pump function in cell membrane was greatly reduced by 5% ethanol treatment.

Under the inhibition of ABCG2 function by 5% ethanol, cytotoxic DDP could stay in lung SP cells as well as non-SP cells and thus efficiently kill them. Our results show that DDP in 5% ethanol (5% ethanol–DDP) could kill both lung SP cells plus non-SP cells *in vitro* with FACS analysis and DDP-resistant tumor cells *in vivo *with TUNEL assay. Intratumoral injection of 5% ethanol–DDP could induce complete tumor regression in 30% of the mice by killing lung SP cells plus non-SP cancer cells and significantly improved tumor-bearing mice survivals.

5% Ethanol–DDP could eradicate tumors by killing lung SP cells plus non-SP cancer cells, presenting the strongest *in vivo* effects among all the reported CSC-targeting agents in solid cancers (Niu et al., 2012; Sachlos et al., 2012; Vermeulen et al., 2012; Visvader and Lindeman, 2012; Zhang et al., 2012; Okuda et al., 2013). By contrast, other investigated anti-CSCs agents including low molecular weight heparin, metformin, dopamine receptor antagonist, mithramycin, salinomycin, sulforaphane, miR-34a, and CSC-specific signaling pathway inhibitors have mostly attenuated rather than eradicated solid tumors in preclinical models by targeting only fixed population of CSC-like cells. And unharmed cancer cells may dedifferentiate into CSC-like cells later. These suggest that targeting both lung SP cells and non-SP cancer cells by 5% ethanol–DDP is superior to other agents designed to target fixed population of CSC-like cells since cancer cells can also dedifferentiate into CSC-like cells (Niu et al., 2012; Sachlos et al., 2012; Vermeulen et al., 2012; Visvader and Lindeman, 2012; Zhang et al., 2012).

Also, survival is the most important parameter reflecting effects of CSC-targeting agent. However, previously studied CSC-targeting agents did not show their survival benefits in solid cancers except for miR-34a (Liu et al., 2011; Visvader and Lindeman, 2012). But, only six mice and 13 days of observance after treatment start in Liu’s study could not sufficiently show miR-34a’s survival benefits (Liu et al., 2011). By contrast, after 180 days’ observance, our study showed that 80% (8 of 10 mice) of 5% ethanol–DDP-treated mice were still alive while all 10 control mice died before Day 123. The dramatic survival advantage in our study indicates that targeting both SP cells and non-SP cancer cells in the tumor by 5% ethanol–DDP has clear advantage over other agents designed to specifically target fixed population of CSC-like cells.

High concentrations of ethanol (more than 95%) alone has been successfully used in various types of tumor ablation especially in liver tumor less than 3 cm during past 20 years (Ellman et al., 1981; Livraghi et al., 1986; Kuang et al., 2009). Although high concentrations of ethanol can destroy various types of smaller tumors than 3 cm through inducing tumor cells dehydration and necrosis, proteins degeneration in larger tumor tissue induced by ethanol can result in boundary formation, which impedes ethanol or toxic agents to diffuse fully in the whole tumor and leads to some areas unharmed (Lee et al., 1995). Also, higher concentration of ethanol can cause severe damage to normal tissues like lung and intolerable side effects to cancer patients, limiting its clinical applications (Nair et al., 2008).

Studies by Pietronigro et al. (2003) showed that intratumoral injection of chemotherapeutic agent BCNU dissolved in 100% ethanol could produce a 40% cure rate in rats bearing intracranial T9 tumors and 72% stable disease in recurrent malignant glioma patients (Jenkinson et al., 2010). However, our unpublished data showed chemotherapeutic agent DDP when dissolved in high concentration of ethanol such as 50% ethanol produced minimal tumor inhibition associated with increased tumor angiogenesis with obvious normal tissue damage compared with 5% ethanol–DDP. This is consistent with Tan and Forsyth’s results that ethanol could stimulate angiogenesis and promote tumor growth (Tan et al., 2007; Forsyth et al., 2010). Together with boundary formation effects and intolerable side effects of 100% ethanol, 5% ethanol shows its superiority over high concentration of ethanol as a solvent of cytotoxic chemotherapeutic agents such as cisplatin or BCNU to destroy tumors.

Since ethanol and DDP are cheap and safe drugs available in clinic for decades, local administration of them in minimal dosage has clear advantage over other investigated CSC-targeting agents on the bench to improve lung cancer survival in clinic.

This study reports that 5% ethanol–DDP produced the most potent *in vivo* effect among all reported CSC-targeting agents by eradicating DDP-resistant lung tumors and extending survivals. Since both ethanol and DDP have been used safely in clinic for decades, image-guided intratumoral injection or intrapleural infusion of 5% ethanol–DDP to treat chemo resistant lung tumor or malignant pleural effusion might provide a novel way to improve lung cancer survival in clinic.

In conclusion, our studies showed that 5% ethanol decreased ABCG2 protein function and 5% ethanol–DDP induced apoptosis of lung SP cells plus non-SP cancer cells *in vitro* and DDP-resistant lung tumor cells *in vivo*. Intratumoral injection of 5% ethanol–DDP could eradicate DDP-resistant lung tumors and extend survival, providing a novel approach to improve chemo resistant lung cancer survival.

Clearly, clinical studies are needed to elucidate the efficacy and safety of this novel approach.

## MATERIALS AND METHODS

### ETHICS STATEMENT

Animal experiment was conducted with the approval of the Institutional Animal Care and Use Committee of the China Agricultural University. The conditions of the animals were monitored daily for evidence of illness. Four steps were taken to minimize the suffering of the animals as follow. Firstly, air exchange, temperature, humidity, noise, light intensity, and light cycles were maintained within limits compatible with the health and well-being of the mice. Secondly, cleaning practices were monitored on a regular basis to ensure effective hygiene and sterile sanitation. Thirdly, to avoid unnecessary harm, drugs were injected gently. Fourthly, mice showing severe distress including infection, ulceration, cachexia, inability to ambulate, and moribund were euthanized humanely in accordance with animal care protocol. The euthanasia by cervical dislocation was used to sacrifice mice. All mice were allowed to die after 180 days’ observance. Two mice which showed severe distress because of biting infection in tumors died for non-tumor-related reasons.

Some mice in this study suffered big tumors because the survival research requires an observance time as long as possible. Meanwhile, we previously found that mice receiving ethanol treatment showed larger tumors and better general conditions compared with control mice. As a result, mice with larger tumors still looked and lived well in general conditions even 3 weeks before severe distress appeared.

### CANCER CELL CULTURE AND REAGENTS

The cisplatin-resistant human lung adenocarcinoma cell line A549/DDP was obtained from the American Type Culture Collection (Rockville, MD, USA) and routinely cultured in RPMI medium supplemented with 10% fetal bovine serum (Invitrogen China Limited, Beijing, China) and 2 μmol cisplatin (Qilu Pharmaceutical Co., Ltd., Shandong, China). A549/DDP cells were cultured in complete RPMI medium without cisplatin for 3 days before being used in experiments. >99.9% ethanol (vol/vol) was bought from Sinopharm Chemical reagent Company (Beijing, China). 5% ethanol (vol/vol) was prepared with >99.9% ethanol and sterile water.

### SIDE POPULATION SORTING AND ABCG2 FUNCTION ANALYSIS

A549/DDP cells were suspended at 1 × 10^6^ cells/ml in phosphate buffered saline solution (PBS) containing 4% FBS, incubated at 37°C for 60 min with 5 μg/ml Hoechst 33342 (Sigma, St. Louis, MO, USA) either alone or in the presence of 500 μmol verapamil (Sigma, St. Louis, MO, USA). After incubation, 1 μg/ml propidium iodide was added and then the suspension was filtered through a 40 μm cell strainer (BD Bioscience, San Diego, CA, USA) to obtain a single cell suspension. Flow cytometry analysis and sorting were performed using a FACS Vantage SE (BD Bioscience). Hoechst 33342 was excited with the UV laser at 350 nm and fluorescence emission was measured with 405/BP30 (Hoechst blue) and 570/BP20 (Hoechst red) optical filters. A549/DDP cells treated with 5% ethanol or control medium for 45 min were investigated for ABCG2 function by analyzing their Hoechst extrusion properties and their sorting rates of SP cells were determined.

### QUANTITATIVE REAL-TIME RT-PCR

5% ethanol or control treated A549/DDP SP cells (for 2 h) and tumor cells collected from 5% ethanol-treated or control mice were suspended at 1 × 10^6^ cells/ml in 5 ml PBS as sample respectively. Then, isolation of total RNA was performed using RNeasy Mini Kit (Qiagen, CA, USA) according to manufacturer’s instructions. The mRNA levels were analyzed by quantitative RT-PCR using a Bio-Rad iCycler system (Bio-Rad, Hercules, CA, USA). The mRNAs were reverse-transcribed into cDNAs by using an iScript cDNA synthesis kit (Bio-Rad, Hercules, CA, USA). The primer sequences and reaction conditions are same with those in previous studies (Niu et al., 2012).

### WESTERN BLOTS

Side population cells were treated with 5% ethanol or control medium for 2 h and harvested for western blots analysis. Western blots were carried out using the protocol published by Niu et al. (2012). Primary antibody incubation was carried out at 4°C for 16 h with a mouse anti-ABCG2 antibody (Biolegend, San Diego, CA, USA) diluted at 1:300 in 3% bovine serum albumin, followed by incubation with horseradish peroxidase–conjugated horse anti-mouse secondary antibody, and developed using an enhanced chemiluminescence detection system (GE Healthcare, Piscataway, NJ, USA). Anti–β-actin antibody was used as the loading control (Santa Cruz Biotechnology, Santa Cruz, CA, USA). The densities of the protein bands in western blot were quantified with the Quantity One-4.6.2 program (Bio-Rad, Berkeley, CA, USA).

### ATPase ACTIVITY ASSAYS

1 × 10^6^ SP cells were treated with 5% ethanol or control medium for 2 h, and then thrice frost thawing was performed for cell disruption. The total protein was determinated by the method of Bradford. The ATPase activities were assayed by the quantization of phosphonium ions; the assay was performed in accordance with the ATPase detection protocol developed by the Nanjing Jiancheng Bioengineering Institute (Nanjing, China; Wang et al., 2008). The reaction product was tested by 721-spectrophotometer at the wavelength of 660 nm. ATPase activity of A549 SP cells (μmol Pi/mg protein/h) were calculated as the quantity of Pi (μmol), which decomposed from ATP by 1 mg ATPase in 1 h.

### TRANSMISSION ELECTRON MICROSCOPY

For TEM, 1 × 10^6^ SP cells were treated with 5% ethanol or control medium for 2 h and then were fixed for 3 h with buffered 1% parahormaldehyde–1.25% glutaraldehyde, osmicated, dehydrated, and embedded in Epon. Slips were examined by transmission electron microscopy JEM-2010 (JEOL, Tokyo, Japan) and FEI Tecnai 20 (FEI, Eindhoven, Netherlands).

### APOPTOSIS ASSAYS BY FLOW CYTOMETRY

2 × 10^6^ A549/DDP SP cells or 2 × 10^6 ^normal A549/DDP cells were treated with 5% ethanol, 5% ethanol plus 2.2 μg/ml cisplatin (DDP), DDP, and control for 2 h and then stained using the Annexin V-FITC apoptosis detection kit (BioVision, Mountain View, CA, USA) and analyzed on a FACScalibur flow cytometer (BD Bioscience).

### TUNEL APOPTOSIS ASSAYS OF TUMOR TISSUES

Briefly, dewaxed tissue sections were predigested with 20 mg/ml proteinase K for 20 min and incubated in PBS containing 3% H_2_O_2_ for 10 min to block the endogenous peroxidase activity. After blocking endogenous peroxidases, tissue sections were incubated in equilibration buffer and treated with terminal deoxynucleotidyl transferase (TdT) enzyme to detect TUNEL-positive nuclei according to the manufacturer’s guide (Roche Scientific, Indianapolis, IN, USA). Tissues were then incubated with converter-POD (Roche Scientific, Indianapolis, IN, USA) and color developed with diaminobenzidine (DAB; Sigma-Aldrich, Munich, Germany). After counterstaining with methyl green, sections were protected with cover slip and secured with mounting medium. Positive control sections were incubated with 5 μg/ml DNase (Sigma-Aldrich, Munich, Germany) for 10 min at 37°C to induce DNA strand breaks. Negative control sections were obtained by omitting the TdT enzyme from the TdT reaction buffer.

### TUMOR GROWTH XENOGRAFT STUDY

Six-week-old inbred male Balb/C nude mice were obtained from the Institute of Chinese Association for Laboratory Animal Science (CALAS, Beijing, China). A549/DDP lung adenocarcinoma cells (5 × 10^6^) were subcutaneously inoculated in the upper left flank on day 1. When the diameters of tumors were >5 mm, the mice were divided into control, 5% ethanol, DDP, 5% ethanol + DDP (*n* = 10 each). Mice in each group were treated with intra-tumoral injection of 150 μl of sterile water, or 5% ethanol, or 8 mg/kg DDP in sterile water, or 5% ethanol + 8 mg/kg DDP accordingly twice a week for 4 weeks. All solutions used sterile water as solvent. Tumor volume was determined with caliper and calculated using (width^2^ × length)/2 twice a week. Survivals of mice in each group were observed and recorded (Niu et al., 2012). To minimize the suffering of the animals, cleaning practices were monitored on a regular basis to ensure effective hygiene and sterile sanitation. To avoid unnecessary harm, drugs were injected gently. The conditions of the animals were monitored daily for evidence of illness. Mice showing severe distress including infection, ulceration, cachexia, inability to ambulate and moribund were euthanized in accordance with animal care protocol. All mice were allowed to die after 180 days’ observance.

### STATISTICAL ANALYSIS

Data are presented as mean ± SD except for survival analysis as mean ± SE. Paired and unpaired student’s *t*-test was used to analyzing two groups of paired or unpaired data, respectively. Repeated measured analysis of variance (ANOVA) was used for comparison of multiple groups. Survival analysis was calculated according to the Kaplan–Meier method and log-rank (mantel–cox) test with SPSS software (IBM, Armonk, NY, US). Mice alive at the end of the study were censored. Differences were considered significant at *p *< 0.05.

## Conflict of Interest Statement

The authors declare that the research was conducted in the absence of any commercial or financial relationships that could be construed as a potential conflict of interest.
